# Training-Schools and Public Health Work

**Published:** 1918-07-06

**Authors:** 


### THE MATRONS' AND SISTERS' DEPARTMENT.

### TRAINING-SCHOOLS AND PUBLIC HEALTH WORK.

The training afforded to nurses in hospitals is recognised as very desirable for women undertaking municipal posts such as those of health inspector, health visitor, welfare superintendent, and the like. In certain districts the municipal authorities give higher salaries to women who have obtained training of this description. But so far there is no general move in the direction of making nursing certificates indispensable. There are still many health visitors who have no experience of nursing, many health inspectors who know nothing by actual experience of the exact science of hygiene as practically exhibited in hospitals. The reason for this anomaly is that the nurse-training schools have hitherto strangely ignored the needs of the National Health Service. It is accepted as an axiom that nurses are to be trained for tending the sick, not for keeping the community free from sickness. The one great purpose cherished by Florence Nightingale in the development of the Nursing Service is forgotten. She certainly did not deem it to be essential to the nurse's function to wait until people fell ill from neglect of sound principles before beginning her duties.

The third session of the Nursing Conference of the College of Nursing, which dealt with nurses and State service, was a little disappointing from this point of view. It did not shed light on the manner in which the training-schools might meet the needs of the municipal authorities. No training-school (we are open to correction if we err) gives facilities to its probationers to study public health, or to pass during their term in hospital any of the examinations recognised as the passport to municipal work. After taking three or perhaps four years' work at her hospital the nurse has to begin her training for public work from the beginning. There has been no attempt to lead her inclinations towards State or municipal service. On the contrary, even supposing she started her training with the design of taking up health work later on, the probability is there will be much to weaken her resolution in the course of her training, so resolutely do the hospital authorities cut her off from all larger issues during the semi-conventual existence to which she is vowed while in training.

Yet the larger hospital training-schools, were they a little more elastic in method, have at their command ample facilities for training a group of nurses in such a way that, on the completion of four years' work, they could at once pass on to those lower posts under trained officials where alone public health training may be adequately completed.

During the probationer's first year in hospital there need be no differentiation between the health nurse and what may be called the disease nurse. But by the middle, at any rate, of the second year there might begin to be a change. For the nurses preparing for public health work experience in the children's wards, for instance, in tuberculosis, or in other directions would be more valuable than acute surgical work. The out-patient and casualty departments would advance her education better than the operation theatre and so on. Lectures in preparation for one or other of the examinations necessary for advancement in her career might supplement or possibly even take the place of the usual courses on medical and surgical nursing, and if these could not be arranged in the institution facilities for attendance on outside lectures might be given, the note-taking and preparation being under the supervision of the sister-tutor, who would direct the entire course of study. In the third or fourth year the nurses might have a term of service with the hospital almoner, and might be trained to follow up cases and learn the workings of the system. It may be objected that this kind of training would withdraw the probationer from the wards, but this objection arises out of a narrow view. Nurses preparing seriously for public posts would be in the same position towards the hospital as midwifery pupils or students of remedial exercises. They would gladly pay special fees in order to profit by the mass of material in preventive work which the hospitals, rightly organised, could afford. We gave some account a short time back of the volume of work accomplished in this direction by the almoner's department at St. George's. It only needs a constructive policy to make the larger hospitals the recognised training centres for women undertaking municipal duties. That can never come about until there is a distinct course for such women, comprising preparation for all the examinations they need to pass in order to enter the public service.

